# Herbivory and Body Size: Allometries of Diet Quality and Gastrointestinal Physiology, and Implications for Herbivore Ecology and Dinosaur Gigantism

**DOI:** 10.1371/journal.pone.0068714

**Published:** 2013-10-30

**Authors:** Marcus Clauss, Patrick Steuer, Dennis W. H. Müller, Daryl Codron, Jürgen Hummel

**Affiliations:** 1 Clinic for Zoo Animals, Exotic Pets and Wildlife, Vetsuisse Faculty, University of Zurich, Zurich, Switzerland; 2 Institute of Animal Science, University of Bonn, Bonn, Germany; 3 National Park ‘Bavarian Forest’, Grafenau, Germany; 4 Florisbad Quaternary Research, National Museum, Bloemfontein, South Africa; 5 Ruminant Nutrition, Department of Animal Sciences, University of Göttingen, Göttingen, Germany; University of Pennsylvania, United States of America

## Abstract

Digestive physiology has played a prominent role in explanations for terrestrial herbivore body size evolution and size-driven diversification and niche differentiation. This is based on the association of increasing body mass (BM) with diets of lower quality, and with putative mechanisms by which a higher BM could translate into a higher digestive efficiency. Such concepts, however, often do not match empirical data. Here, we review concepts and data on terrestrial herbivore BM, diet quality, digestive physiology and metabolism, and in doing so give examples for problems in using allometric analyses and extrapolations. A digestive advantage of larger BM is not corroborated by conceptual or empirical approaches. We suggest that explanatory models should shift from physiological to ecological scenarios based on the association of forage quality and biomass availability, and the association between BM and feeding selectivity. These associations mostly (but not exclusively) allow large herbivores to use low quality forage only, whereas they allow small herbivores the use of any forage they can physically manage. Examples of small herbivores able to subsist on lower quality diets are rare but exist. We speculate that this could be explained by evolutionary adaptations to the ecological opportunity of selective feeding in smaller animals, rather than by a physiologic or metabolic necessity linked to BM. For gigantic herbivores such as sauropod dinosaurs, other factors than digestive physiology appear more promising candidates to explain evolutionary drives towards extreme BM.

## Introduction

### 1.1 Reconstructing dinosaur feeding behaviour and trophic niches

Dinosaur gigantism, in particular in its spectacular form of the sauropod dinosaurs, has fascinated scientists for centuries [1]. Sauropods dominated terrestrial ecosystems for more than a hundred million years [1]. Coupled with this evidence of ecophysiological success, their existence raises the question what factors selected for their very large body size? Among the various possible answers, advantages in digestive physiology bestowed by large body size have been suggested [1]. This review will examine the role of digestive physiology as a driver for increasing body mass in herbivores by reviewing evidence accumulated from studies of contemporary herbivores.

There are generally two ways to reconstruct dinosaur feeding behaviour, trophic niches and digestive physiology: using morphological characteristics of the cranium, the neck or even the whole body, and using (quantitative and qualitative) extrapolations based on body mass (BM). Differences in skull anatomy, dentition, neck height and position, tooth microwear and stable isotope composition between different sauropod clades have been presented and used to evoke niche separation and differential resource use in different and also in sympatric sauropod species [2]–[11], and are not reviewed here. The second option – reconstructions by extrapolating from extant animals, based on relationships between BM and diet quality, diet selection, and digestive physiology - has also been used extensively in reconstructing dinosaur physiology [12]–[14] and is the topic of this review.

### 1.2 The use of allometries

Dealing with extrapolations based on BM, one usually refers to allometric relations that are described by the equation y = *a* BM*^b^*. Usually, *b* is different from 1, i.e. the relationship is not linear (i.e., does not follow the ‘same measure’ in ‘iso-metry’) but follows ‘another measure’ (hence the term ‘allo-metry’). If *b* is smaller than 1, the measure, expressed in % of BM, will decrease with increasing BM. This relation is sometimes also referred to as a ‘lower mass-specific measure with increasing BM’. In the scientific literature on allometries, the (exact) magnitude of the exponent is often an important part of a concept, such as in the metabolic theory of ecology [15]. In this review, we mostly refrain from citing or analysing the magnitude of the exponent unless it is necessary for the argument. We do this to avoid confusion, because the different published allometric exponents were derived with considerable discrepancy between publications, both in terms of the species set used (which may, for example, include mammals, or only mammalian herbivores, African mammalian herbivores, ruminants, grazing ruminants etc.), and in terms of the methods employed (which may or may not include the use of one mean value per species or log-transformation prior to model fitting, or account for the phylogenetic structure of the dataset etc.). For example, scaling exponents can vary significantly depending on whether the phylogenetic structure of the data is accounted for or not [16], [17]. Another important problem in comparing allometries is that the compatibility of the different measures that all scale to BM must be given [18]. If we use, for example, faecal nitrogen as a proxy for diet quality, and assume that a 10 kg animal has values of 4% nitrogen in the organic matter of the faeces (OM), and a 3000 kg animal 0.8%OM (which is roughly the range covered in [18]), the resulting allometric scaling exponent for diet quality would be BM^−0.28^. If we use these faecal nitrogen values, however, to calculate organic matter digestibility of the diets (using the curvilinear regression equation of Lukas et al. [19]), the resulting values are 77.6% and 29.5% for the small and the large animal, respectively, yielding a scaling of BM^−0.17^. The question which of the two scaling exponents should be used in further calculations is difficult to answer, but mixing them or using them to frame a range of options is akin to lumping length measurements taken in centimetres and inches. Ideally, all measures used in such allometry-based concepts should be linked in a logical, physical (and hence mathematical) way, as for example food intake, retention time of digesta in the gastrointestinal tract, digestibility and gut fill that are linked via a physical principle [16], [20]. All these difficulties make comparisons of different allometric exponents from different publications unreliable, unless they are controlled for in a single analysis. We will mention several methodological aspects of using allometries in the text below (see also [21]).

One of the most important misunderstandings when dealing with allometries [22] shall, however, be mentioned here already; it is for example evident when citing the following passage from Geist [23] explaining the Jarman-Bell-principle: *‘The daily energy and protein requirements of mammals are a function of their body weight raised to the power of 0.75. For this reason, small-bodied species require more energy and protein per day per unit of body weight than do large-bodied forms (assuming identical work regime and exposure to temperature and wind). The high metabolism of small-bodied species can be sustained only on highly digestible forage. Since digestibility, and hence daily intake of forage, is a function of the fiber and protein content of the forage, small-bodied ungulates require a forage of relatively low fiber content and high protein content; large-bodied ungulates can feed on forage with higher fiber and lower protein content since their requirement for energy and nutrients per unit of body weight are lower.’* Presented like this, this argument has no power as the scaling of a single measure (here, energy requirement) in itself explains nothing. Only when compared against a scaling of another measure (such as intake or intake capacity) do further deductions become feasible. The expression of the allometric relationship as ‘smaller species requiring more per unit body weight’, while mathematically correct, would only explain anything if it was shown that some other factor relates directly to ‘unit body weight’. The statement that smaller animals ‘have higher mass-specific metabolic requirements than large animals’ expresses the same fact as the statement that smaller animals ‘have the same metabolic requirements as large animals on a metabolic body weight basis’ (note that the allometric relationship also allows to correctly state that ‘smaller animals have lower absolute metabolic requirements than large animals’). In the scenario outlined in the citation, one can only conclude that

- Requirements scale to BM^0.75^, so the intake of a specific diet should scale to *a* BM^0.75^.
- Animals faced with a lower-quality diet will have to eat more of this diet (this is valid for animals *of all size classes*). Intake of this diet will therefore scale to *c* BM^0.75^, where *c*>*a*.
- Animal faced with a higher-quality diet will have to eat less of this diet (again, this is valid for animals *of all size classes*). One could assume that intake of this diet should therefore scale to *d* BM^0.75^, where *d*<*a*.

Other conclusions are not valid based on the citation alone. In particular, the single scaling can give no compelling reason why a certain size class requires a different diet quality than another. Evidently, if intake capacity could be shown to be constrained in smaller animals, so that reaction b) was not possible, or if encounter rate was constrained in larger animals so that reaction c) was not possible, this would have great explanatory power. But the words ‘higher mass-specific requirements’ do not represent such evidence.

## Concepts of Herbivore Body Size and Diet Quality

### 2.1 Body size and food abundance

**Figure 1:**
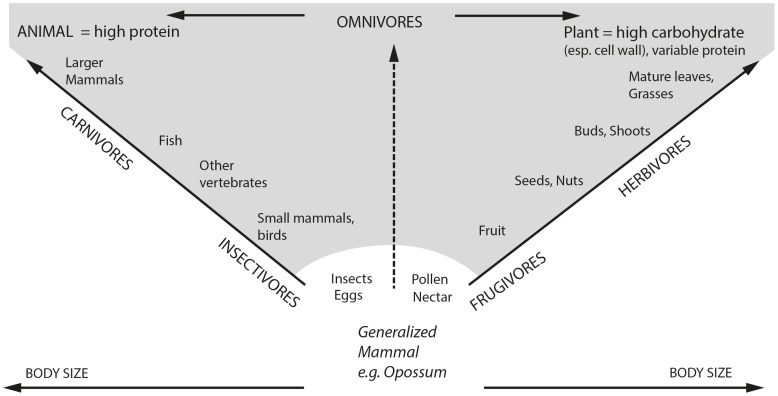
The link between body size and availability of prey in sufficient amounts/packages in terrestrial vertebrates. Modified from Hiiemae [131]. Note that large body size is linked to prey (package) abundance and accessibility, not necessarily to low diet quality per se.

We think that in general, there is consensus that herbivores of higher BM ingest diets of lower quality. This is due to the fact that larger animals require larger quantities of food, yet in terrestrial ecosystems, the more abundant plants and plant parts (such as stems or twigs) are generally of lower nutritional quality than less abundant, higher-quality parts (such as leaves or fruit) [24]; note that this applies to both browse and grass forage. This observation is part of a general concept that links the diets of animals to the abundance of their food (Fig. 1), and both large carnivores and large herbivores have to focus on those food items of which they can find sufficient amounts of accessible packages to satisfy their requirements – in herbivores, this is abundant low-quality forage, in carnivores, large (and high-quality) vertebrate prey [25], [26]. Because of basic geometry, and also in order to meet their high absolute food requirements, the feeding apparatus of larger species is often of a dimension that in itself prevents selective foraging in terms of both, selecting of small, high-quality plant species, and selecting high-quality plant parts [27], [28]. Thus, on land, large herbivore BM will most likely imply a low quality diet because of biomass availability and the ability to feed selectively, but it does not physiologically oblige animals to consume such diets if higher quality food is available in reasonable amounts. In the marine environment, where high-quality food exists in spatially and temporally aggregated lumps of krill or fish that can be easily harvested, gigantism occurs in conjunction with this high-quality food ([29]; note that the lower-quality primary production - algae - is of a dimension that makes it unfeasbile for harvest by larger organisms).

**Figure 2:**
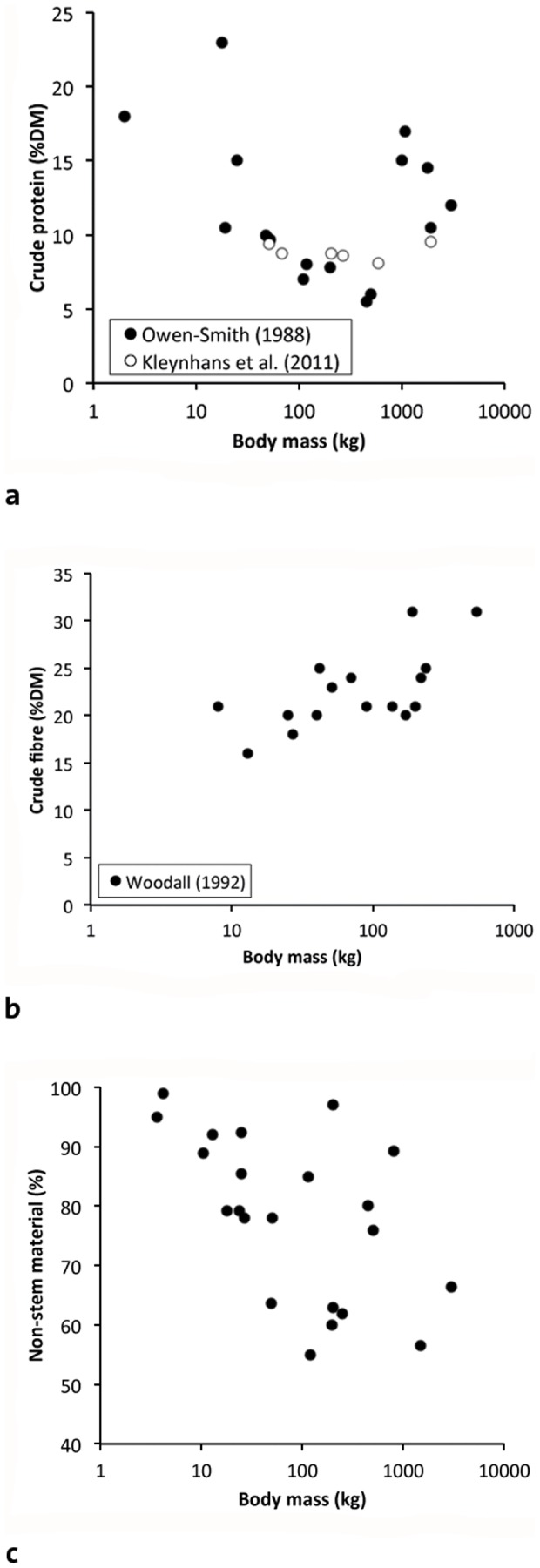
Relationship between herbivore body mass (BM) and characteristics of the natural diet that are indicators of diet quality from comparative studies in African mammals. a) BM and nitrogen concentration in (fore)stomach contents [42] or the measured diet [43]; note that large herbivores (giraffe, rhinos, hippo, elephant) oppose the trend in the smaller species; b) BM (estimated from other sources) and the crude fibre concentration in rumen contents (data on ruminants only) [52] ; c) BM and the proportion of non-stem material in the rumen [42], [53], [91], [133]–[140]; note that browsing ruminants of very small (dikdik), small (duiker, steenbok), intermediate (bongo) and large size (giraffe) show less systematic variation with BM, but their selective inclusion/exclusion will influence the data set; note also that the African buffalo (and also the hippo) do not follow the clear negative trend seen in smaller grazers.

**Figure 3:**
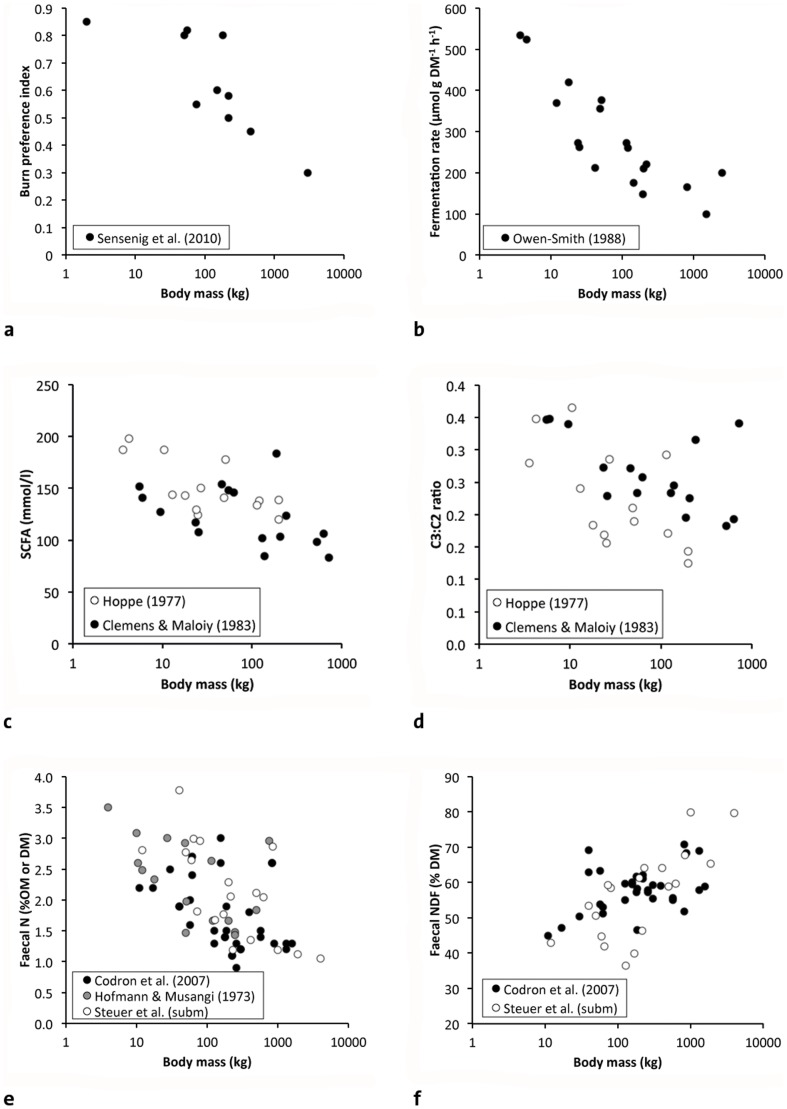
Relationship between herbivore body mass (BM) and characteristics of the natural diet that are indicators of diet quality/degradability from comparative studies in African mammals. a) BM and the preference for newly burned savanna patches from Sensenig et al. [55] (note that the study did not include rhinos or hippos); b) BM and in vitro fermentation rates (a proxy of microbial digestion) in rumen, forestomach (hippo) or caecum (elephant) contents [42]; c) BM and the concentration of short-chain fatty acids (SCFA, which represent products of microbial digestion) [135], [141]; d) BM and the ratio of the SCFA propionate (C3) to acetate (C2) (a proxy of the proportion of easily fermentable carbohydrates in the diet) [135], [141]; e) BM and nitrogen content of faeces (a proxy for diet digestibility; [18] – organic matter OM basis, [142] - OM basis, [143] – dry matter DM basis); f) BM and the neutral detergent fibre (NDF) content of faeces [18], [143].

Nevertheless, actual proofs of the relationship between herbivore BM and diet quality are rare in the scientific literature (see below). Most comparative datasets on this topic represent studies on African savannah systems (Fig. 2 and 3), but the clarity of the result often depends on the assemblage of species, feeding types (grazing/browsing) and digestion types (ruminant/hindgut fermenter) used. In combinations of small browsing ruminants, grazing ruminants of all sizes, and hindgut fermenters in the ruminant size range (warthog, zebra), trends of decreasing diet quality with increasing BM are mostly evident. If, however, additional species are included in the dataset, such as large browsing ruminants, rhinoceroses, hippopotamus, and elephant, these latter species often oppose the clear trend observed in the other species (see below), which evidently has important implications for any concept that links body size and diet quality. One of these implications is that differences in organismal design can blur patterns related to BM only [16] – which might make simple relationships with BM questionable in the first place.

### 2.2 Setting the question: Can low food quality drive body size evolution?

The observation of the association of large BM and low diet quality allows the following (non-exhaustive) combinations of hypotheses

1. Low diet quality is an unavoidable consequence of large herbivore BM and
2.
  - large BM provides advantages that specifically enhance the use of low quality diets or
  - large herbivores have to (and evidently can) cope with low quality diets without being endowed with specific advantages linked to their large BM.

The important difference between hypothesis 2a and 2b is that if 2a is true, then we could postulate selective pressure for larger BM and even gigantism by paleoenvironments in which diets were of inherently low quality [14], [30]; if 2b is true, then other factors must have driven evolution towards gigantism. In the literature on species diversification and niche differentiation of extant large herbivores, it is widely assumed that *‘size itself is an important adaptation, because the effect of lower selectivity in large animals would appear to be easily outweighed by their greater digestive efficiency and fasting endurance’* (p. 85 in [31]), supporting hypothesis 2a.

## Characterising Diet Quality and Herbivore Adaptations

In order to investigate these hypotheses, we need to use different definitions of how ‘low diet quality’ can be quantified. With respect to the most often cited criteria for low diet quality, we differentiate between

a high content of plant secondary plant metabolites such as tannins (e.g. [30]),
a low content of protein (measured as nitrogen, and also expressed as the carbon∶nitrogen [C∶N] ratio) [14], [30],
a high content of slowly digestible and/or indigestible fibre components such as (hemi)cellulose or lignin [24]
and finally a generally low ‘digestibility’ – a measure all three previous measures, but especially cellulose and lignin, are linked to.

When investigating the effects of these properties, we require both logical concepts (why they are a consequence of large herbivore BM and why large BM might represent an adaptation to them), and empirical data supporting these concepts.

### 3.1 Diet quality: Plant secondary metabolites

To our knowledge, no empirical evidence exists that larger herbivores ingest diets that have higher contents of plant secondary metabolites (PSM). However, it has been postulated that larger herbivores need to reduce the level of any specific PSM, assuming that their lower mass-specific metabolic rate is also linked to a generally lower detoxification metabolism [32]. In an analysis of the feeding records of 74 animal species, Freeland [32] demonstrated that the number of plant species included in a natural diet increases with BM, thus limiting the proportion of a single species within the total diet. A wider range of different forage species is commonly associated with a wider range of different PSM, and dietary variety is therefore commonly interpreted as a strategy to avoid the accumulation of any one particular PSM to toxic levels (e.g. [33], [34]). Therefore, Freeland [32] hypothesized that the body size-diet variety relationship exists because small animals can detoxify larger amounts of a particular plant toxin and thus do not need to show the same degree of dietary variety as larger animals. According to this logic (which we do not accept, see below), higher levels of PSM would prevent the evolution, or drive the extinction, of larger BM. In line with this concept, Guthrie [35] hypothesized that a reduction in available plant variety causes the decline of very large species, a case he exemplifies with the well-recorded decline in variety of diet that preceded the extinction of the Shasta ground sloth (*Nothrotheriops shastense*). To our knowledge, no association between plant variety and dinosaur gigantism was made to date in corresponding analyses for dinosaurs (e.g. [36]).

The logic of the detoxification-rate argument requires closer scrutiny. The statement that larger animals have ‘lower mass-specific metabolic rates’ (i.e., lower metabolism per unit BM) is true, yet explains nothing – the scaling of one single parameter in itself has no explanatory power unless it is related to the scaling of another parameter (cf. section 1.2). Even if detoxification metabolism were linked to overall metabolic rate – a fact that would require empirical support (see below) -, this would only represent a constraint *if PSM intake scaled differently than metabolism*. Note that larger animals also have ‘lower mass-specific food intake rates’ [16]. Basal metabolism of large mammals roughly scales to BM^0.72^ [37]; in larger herbivores, evidence suggests a higher scaling of dry matter intake of about BM^0.84^ [16]. Thus, in theory, if detoxification metabolism for specific toxins scaled in the same way as overall basal metabolism, larger animals might indeed require a more varied diet.

These reflections are contradicted by the finding that folivorous mammals, i.e. mammals which we expect to ingest diets that contain comparatively high amounts of PSM, generally have lower mass-specific metabolic rates than mammal herbivores that consume grass, i.e. lower levels of PSM [38], [39]. This actually suggests not similarity between metabolic and detoxification rates, but a trade-off between the two [40]. PSM elimination has also been associated with mechanisms not directly linked to metabolism, such as the prevention of absorption in the gut [41]. So far, a strict link between overall metabolic rate and mechanisms of toxin avoidance or detoxification has not been presented conclusively. Consequently, the intake of a varied diet will be beneficial for herbivores of any BM, and the relationship between BM and variety mentioned earlier might not reflect a systematic difference of detoxification capacities with BM, but simply the fact that larger animals encounter a higher diversity of plants in their larger home ranges and have to rely on a larger part of the potentially available biomass.

In summary, there is currently no concept that explains why a lower diet quality as defined by higher contents of secondary plant compounds could be a selective pressure for larger herbivore size. The only existing concept even points in the opposite direction, but is not backed by sufficient empirical data.

### 3.2 Diet quality: Protein (nitrogen)

Protein is commonly measured as nitrogen, and we will use the term nitrogen (N) from here onwards. Owen-Smith [42] presented a data collection on the relationship of diet N content (measured in stomach or forestomach contents) and herbivore BM (Fig. 2a). In that data set, there was a negative relationship between ruminant BM and dietary N, supporting the concept of decreasing diet quality with increasing herbivore size in that clade; however, dietary N levels measured for giraffe (*Giraffa camelopardalis*) and large nonruminant herbivores such as rhinoceroses (*Diceros bicornis*, *Ceratotherium simum*), hippopotamus (*Hippopotamus amphibius*) or elephant (*Loxodonta africana*) do not fit the common pattern – a fact that should not be overlooked. This result was repeated in a smaller species set, without elephants but including the white rhinoceros, by Kleynhans et al. [43], where dietary N decreased with increasing BM in the range below 1000 kg, but again with the white rhino as a notable exception (Fig. 2a). As an aside, note that while N levels in stomach contents can be regarded a direct proxy for dietary N, this is not true for faecal N levels (see section 3.4).

Among vertebrates, N requirements of individual species are closely linked to the nitrogen content of their respective diets; thus, carnivores generally have higher N requirements than herbivores, for example [44]. Midgley [45] states that *“herbivore nutritional requirements will evolve in concert with food quality.”* In species with particularly low-N diets, such as nectarivores or gummivores, extremely low N requirements have been demonstrated (e.g. [46]). If faced with a diet of low N content, animals of *any* body size would have to ingest larger quantities of that food to meet their N requirements (see section 1.2), unless they evolved specific physiological traits to reduce N requirements. An adaptive value of large BM in this respect could only be postulated if larger BM facilitated such an ingestion of larger quantities more easily.

Nevertheless, it has been suggested that large body size might represent an adaptation to food of low N content, and hence of a high C∶N ratio [14], [30]. While Midgley et al. [30] do not offer a mechanism by which this might occur but simply refer to the association of large body size and low diet quality, Wilkinson and Ruxton [14] do not only refer to this association, but suggest that this an effect of the discrepancy in the scaling of N requirements and energy requirements with BM. Using published equations on the scaling of N requirements and field metabolic rate for reptiles and mammals from Klaassen and Nolet [47], they calculate a scaling of the ratio of N∶energy requirements of BM^−0.47^ in reptiles (i.e., larger reptiles would require less N per unit energy) and BM^0.09^ in mammals (i.e., larger mammals would require more N per unit energy). Linked with their assumption that large dinosaurs are best represented by extant reptiles, these scaling relationships suggest that low plant N should favour gigantism in herbivorous reptiles (and small body sizes in herbivorous mammals).

This use of allometric reasoning is instructive because of four different deficits. The first three are conceptual. First and most evidently, the discrepancy that for the association of large BM and low diet quality, the study on mammals by Owen-Smith [42] is cited (p. 131 in [14]), yet the results on the scaling of N∶energy requirements in mammals would suggest that larger mammals require particularly high-quality diets (increasing N per unit energy at increasing body size), is not discussed. This discrepancy alone should caution against the use of the N∶energy requirement scaling proposed by the authors.

Secondly, the argument focuses on N as the main indicator of forage quality – in contrast to most other studies in large herbivore ecology (see sections 3.3 and 3.4). Thirdly, the assumption that N requirements could scale differently than energy requirements/metabolism in vertebrates, and in particular in opposite directions in reptiles and mammals, requires a physiological concept, which is not presented. Actually, animal physiologists appear to assume, on the contrary, a scaling of N requirements that is similar to metabolic scaling (BM^0.75^), which would translate into a scaling of N∶energy requirements at BM^0.75^∶BM^0.75^∼BM^0^ (in other words, no scaling). For example, as cited above, Geist [23] stated that *‘energy* ***and protein*** *requirements of mammals are a function of their body weight raised to the power of 0.75’*. In his monograph on ‘Wildlife feeding and nutrition’, Robbins [48] expresses N requirements by default per unit metabolic body weight, or BM^0.75^. When publishing their famous mouse-to-elephant curve that supported the concept of metabolism scaling to BM^0.73^, Brody et al. [49] also reported a mouse-to-cattle curve on endogenous urinary N losses scaling to BM^0.72^, indicating a similarity in scaling of N and energy requirements and, consequently, no scaling (BM^0^) of the ratio of N∶energy requirements. Actually, it is the most parsimonious explanation that all processes responsible for maintenance protein requirements, such as replacement of degraded body protein or enzyme production, are proportional to energy metabolism. Note that the numerical difference between the scaling factors (e.g. 0.73 for metabolism and 0.72 for endogenous urinary N losses in Brody et al. [49]) in itself does not mean much as long as it is not demonstrated that their 95% confidence intervals do not overlap [21].

The fourth concern with this approach relates to the use of empirical data. A closer look at the data from Klaassen and Nolet [47] that resulted in the scaling relationships reported by Wilkinson and Ruxton [14] show that neither author team checked whether the 95% confidence intervals (CI) for the scaling exponents they used overlapped. Using the data supplement from Klaassen and Nolet [47] to calculate these confidence intervals, one notices that the scaling of N requirements in reptiles (at BM^0.473 (95%CI: −2.179;3.126)^, based on a dataset of n = 3 species) is not significant as the 95%CI of the exponent includes zero, and also includes the scaling of field metabolic rate in reptiles (at BM^0.889 (95%CI 0.830;0.948)^ , n = 55 species). For mammals, the 95% CI for N requirement scaling (at BM^0.863 (95%CI 0.769;0.956)^, n = 11 species) and field metabolic rate scaling (at BM^0.772 (95%CI 0.730;0.815)^, n = 79 species) also overlap, again not excluding a similar scaling. Thus, in both cases, a scaling of N∶energy requirements at BM^0^ cannot be excluded, in accord with current physiological theory.

In summary, evidence for decreasing dietary N content with increasing herbivore BM in the range of ungulate herbivores is equivocal so far, but is expected based on the considerations in section 2.1. There is currently no concept that explains why a lower diet quality as defined by lower contents of N could be a selective pressure for larger herbivore BM; current knowledge and data rather support the notion that dietary N content is unrelated to the evolution of BM.

### 3.3 Diet quality: Fibre content

Dietary fibre can be measured in many different ways. In herbivore research, the most commonly used is the system that analyzes acid detergent lignin (ADL; usually considered completely indigestible), acid detergent fibre (ADF; representing ADL plus cellulose), and neutral detergent fibre (NDF, representing ADF plus hemicellulose) by Van Soest [50]. Typically, increasing fibre content decreases overall digestibility, and increasing ADL content in particular reduces fibre digestibility [50]. There is one important difference between these fibre fractions: whereas hemicellulose and cellulose mainly decrease fermentation rate (measured as % per hour) but not necessarily the overall potential digestibility (measured as total %), because they are slowly-fermenting substrates, lignin does not necessarily reduce fermentation rate but does reduce overall potential digestibility, because it is basically indigestible for gut microbes [51].

To our knowledge, only one data collection exists that provides comparative data on the fibre content of (fore)stomach contents, in African ruminants [52]; higher fibre levels in larger ruminants are evident (Fig. 2b). The only other study that gives a proxy for fibre content is again by Owen-Smith [42], who showed that the ratio of foliage∶stem material (i.e., the proportion of non-stem material) in the stomach decreases with increasing herbivore BM, which can be interpreted as an increase in fibre (and a decrease in nitrogen). Re-analysing that dataset for ruminants only, however, and including an additional source for another browsing ruminant of the intermediate body size range (the bongo *Tragelaphus eurycerus* [53]), also allows the interpretation that this ratio mainly separates browsers from grazers. This is also confirmed by the position of the elephant as an intermediate feeder. Hence, any relationship with BM will depend on the selection of browsing species included in the dataset (Fig. 2c); additionally, the hippopotamus does not fit the pattern found in grazing ruminants. In a more recent study, the enormous flexibility of elephants was demonstrated, with the proportion of stems, bark and roots increasing from approximately 30% in the wet season up to 94% in the hot dry season [54]; this wide range indicates that large body size may be linked with the variety of plant parts that can be used, in particular the harder tissues that may be difficult to crop for smaller species. Sensenig et al. [55] showed in a sample of ten African grazing herbivores that the preference for recently burned areas (which contain young regrowth, i.e., plant material of lower fibre and higher nitrogen content than non-burnt patches, but lower standing biomass) decreased with BM (Fig. 3a); notably, neither rhinos nor the hippo were part of that experiment. Results of similar studies with smaller numbers of species suggest that the white rhino would probably be, again, an outlier to this pattern [56], [57]. Using a similar reasoning by deducting forage quality and abundance from climate, geology and landscape indicators, it was demonstrated that herbivore BM distribution followed the distribution patterns expected if larger species require more abundant food (of inherently lower quality) [58]–[60]. Another, similar study showed that larger species were more evenly distributed across habitats than smaller species, corresponding to smaller species relying on spatially less homogenously distributed higher-quality forage [61]; again, the white rhino appeared as an outlier to that pattern. Similarly, the habitat use of three browsing ruminants showed an increasing habitat diversity with body size [62]. White rhinos often (though not always) feed on ‘grazing lawns’, where forage quality is comparatively high due to the regular cropping [63]. By comparison, one would assume that if the hippopotamus, another very large herbivore, would be included in such studies, it would similarly represent an outlier due to a similar feeding behaviour [64].

**Figure 4:**
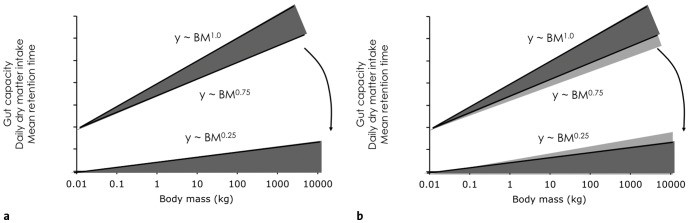
Schematic explanation of circular reasoning in the traditional approach of explaining a positive effect of body mass on digestibility. a) The difference in the scaling of gut capacity (measured as wet or dry gut contents; BM^1.0^) and daily dry matter intake (BM^0.75^), or actual dry matter gut fill rate, results in more gut available per unit digesta at higher BM, and should hence lead to increased mean retention times at higher BM (BM^0.25^). If these increased retention times are used to postulate a higher digestibility at higher BM, the situation in b) occurs: The increasing digestibility reduces the actual gut fill rate, hence increases the difference in the scaling of gut capacity and gut fill rate even more, which should translate into even longer retention times.

These studies all draw on the concept of the ‘fibre curve’, in which it is demonstrated that forage abundance is related to its fibre content, with more fibrous feeds more abundant [24], [65]–[67]. Historically, it has been suggested that large body size confers a digestive advantage in terms of a longer digesta retention time and hence a higher digestive efficiency (reviewed in [16] - see that text for detailed references, and [68], [69]). This concept was repeatedly explained as deriving from a difference in scaling between two digestive parameters: while gut capacity is assumed to scale to *M* ^1.0^, energy requirements and food intake was assumed to scale to *M* ^0.75^. Thus, one would assume larger animals to have a higher gut capacity per unit ingested food, and should therefore have a longer digesta retention time. This should scale at about *M* ^1.0-0.75 = 0.25^ (Fig. 4a). This explanation is explicitly or implicitly used in a very large number of ecological studies, including examples cited above.

This use of allometric reasoning is again instructive because of four different deficits. The first three are again conceptual, of which the first relates to the nature of how forage quality can decline [18]. If lower forage quality is assumed to be mainly characterised by slower microbial fermentation rates, as one would expect by an increasing proportion of (hemi)cellulose, then an increase in retention times could compensate for this phenomenon (by giving gut microbes more time for fermentation). If, however, forage quality is mainly characterised by a lower overall potential digestibility, as one would expect by an increasing proportion of lignin, then increasing retention times would not be of any help, but would actually represent a disadvantage (because indigestible material would just be carried in the gut for a longer period of time) [51]. Thus, the scenario of increasing retention times and digestibility with increasing BM could, if at all, only apply for certain conditions of forage quality decline.

The second conceptual deficit relates to the logic of the scaling derivation: retention time is not only a function of gut capacity and intake, but also of digestibility itself [20], [21]. If digestibility is higher, more food will be absorbed from the digestive tract, will hence not push on along the digestive tract, and hence retention time will be longer (Fig. 4b). When deriving the scaling of retention time from the scaling of gut capacity and food intake, one therefore inadvertently makes an implicit assumption about the scaling of digestibility itself; hence using the resulting scaling to make predictions on digestive efficiency again amounts to circular reasoning [16], [70]. That is unless one also assumes that the increasing digestive efficiency of larger animals exactly outcompensates the decreasing diet quality, and hence leads to no change in the actually achieved digestibility.

**Figure 5:**
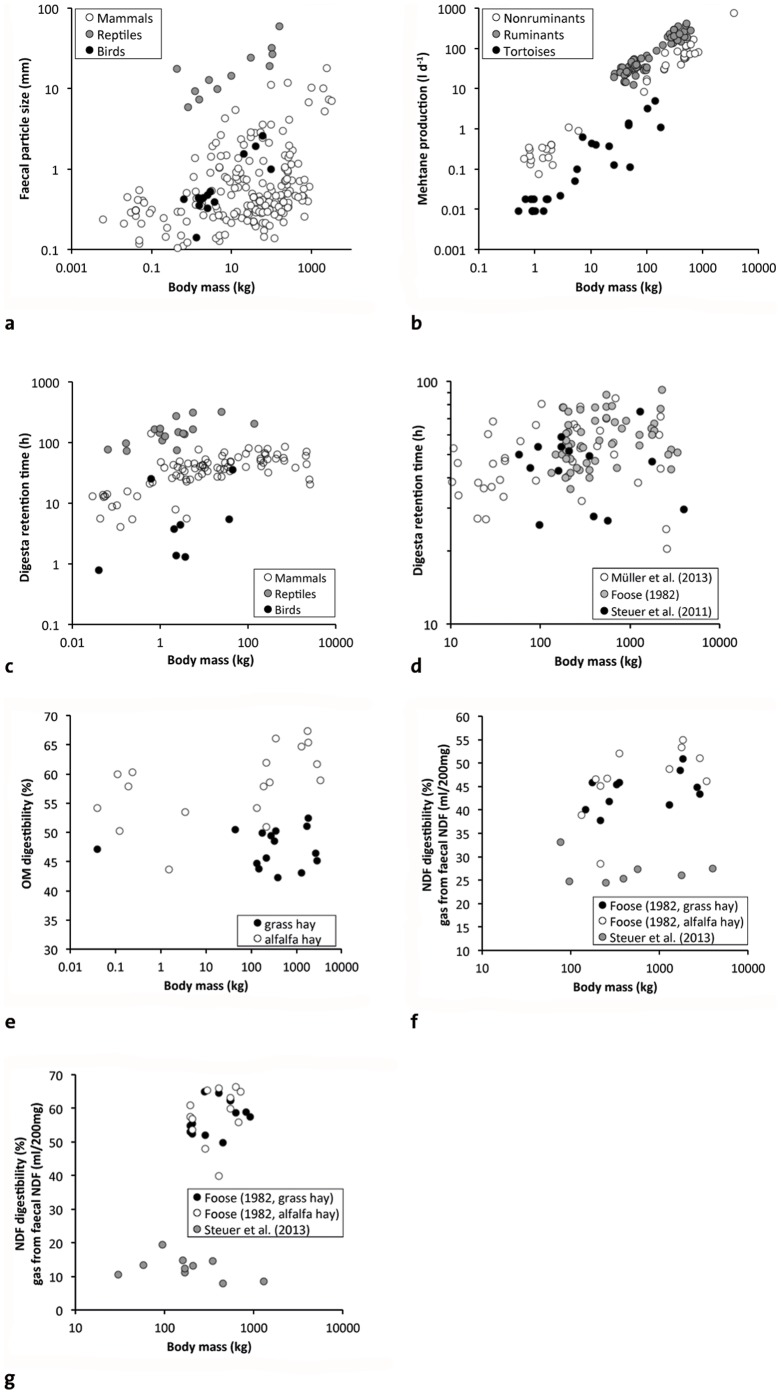
Relationships of body mass (BM) and aspects of the digestive physiology of herbivorous vertebrates. a) BM and faecal particle size in mammal, reptile and avian herbivores [72]–[74]; b) BM and methane production in ruminant and nonruminant mammal herbivores and tortoises (herbivorous reptiles) [75]–[77]; c) BM and particle mean retention time in herbivorous mammals, reptiles and birds [16], [81], [112] (note little increase above BM of 1 kg); d) BM and particle mean retention time in three independent datasets on large herbivorous mammals [16], [66], [80] (note the absence of relevant scaling); e) BM and organic matter digestibility in mammalian hindgut fermenters [71] (note that there is no clear scaling pattern); f) BM and NDF digestibility on two different forages [66] and in vitro faecal NDF gas production (an inverse proxy for fibre digestibility) [86] in mammal hindgut fermenters and g) ruminants (note that there are no clear scaling patterns).

The third conceptual problem is that there are several other animal factors than retention time that have an influence on digestive efficiency [71]. For example, digestion rate is slower for larger particles, and digesta particle size increases with BM in herbivorous mammals [72], reptiles [73] and birds [74] (Fig. 5a). Energetic losses due to methane production appear to increase disproportionately with increasing BM in herbivorous mammals [75], [76] and reptiles [77] (Fig. 5b). These putative digestive disadvantages of large BM would have to be factored into any calculations of the scaling of digestive efficiency with BM.

Finally, empirical data do not match the predicted pattern of longer digesta retention or higher digestive efficiency in larger herbivores above a threshold of about 1–10 kg: digesta retention time does not scale as predicted (dataset from the large comparative study of [66], re-analysed by [16], [42]; analyses of large compiled mammal datasets by [16], [78], [79]; new large comparative mammal study by [80]; compiled datasets herbivorous birds in [81] and on herbivorous reptiles in [82]) but shows a less clear-cut or no relationship with BM (Fig. 5c and d). Correspondingly, there is little indication for a systematic effect of body size on digestibility – neither in compiled datasets [71], [83], [84] (Fig. 5e), in compiled datasets when diet quality was statistically controlled for [82], [85], nor in studies in which the same diets were fed to a variety of species (dataset from the large comparative study of [66], re-analysed by [16], [86]–[88]; new large comparative mammal study by [86]) (Fig. 5f and g). Instead, digestive efficiency appears to be rather independent from BM in these studies.

In summary, although not documented in detail, the association of low diet quality and large terrestrial herbivore size is usually not questioned, but the outlying position of some megaherbivores such as white rhinos or hippos challenge the overall concept. In contrast to a long-standing view of a digestive advantage conferred by large BM in terms of digestive efficiency, neither conceptual nor empirical approaches can support this interpretation.

### 3.4 Diet quality: digestibility/degradability

As already evident in the section above, the term ‘digestibility’ is ambiguous because it usually refers to a measurement (intake minus excretion, divided by intake) in a specific animal (with its species-specific digestive efficiency) on a specific diet (with its diet-specific degradability) in a defined time period (conventionally, 5–7 successive days) [48]. The measure will thus integrate both animal and diet factors. Therefore, other terms like ‘in vitro digestibility’, ‘potential digestibility’ or (here) ‘degradability’ are used to describe the diet-specific component of an actually occurring digestibility [18]. Degradability of a diet represents an integrative measure that is influenced by its fibre, N and PSM content, amongst other factors [51], [89].

Because herbivores rely on symbiotic gut microbes for digestion [90], various proxies of microbial digestion are used to quantify diet degradability. While the degradability can be assessed by in vitro assays, the sampling of the diet itself, as consumed by the animal, is often logistically challenging. For comparative studies, therefore, samples for analysis are commonly taken after the animals performed their diet selection, either by sampling (fore)stomach contents or faeces. Analyses on forestomach contents in herbivores could be assumed to yield similar results as the originally selected forage in in vitro assays, i.e. without a major influence of the digestive efficiency of the animal. However, this assumption might be misleading due to differences in feeding bout intervals and hence the likelihood that sampling was performed on stomach contents consisting of freshly ingested forage or forage that was already subjected to longer microbial digestion. In this respect, the extremely frequent feeding intervals for example in the small dikdik (*Madoqua* spp.) [91] could mean that forestomach contents of hunted animals will always be comparatively homogenous with respect to their digestion state, whereas for example the one nocturnal feeding bout in hippos [92] leads to the risk that forestomach contents of hunted animals may be quite pre-digested before sampling for comparative analyses. In this respect, comparative studies of (fore)stomach contents will provide results that integrate both diet quality and feeding bout frequency.

Microbial digestion is characterised by a fermentation rate: This is commonly measured as gas production in vitro, and was shown to decrease with increasing BM in African herbivores [42] (Fig. 3b). It should be noted that values from the caecum of hindgut fermenters, such as the elephant in this dataset (which already appears as an outlier due to its comparatively high values), are not strictly comparable, because the digesta entering the caecum will necessarily be of a lower quality, due to the preceding digestion in the small intestine, than digesta from the (fore)stomach. Alternatively, one can transform gas production rate into short-chained fatty acid (SCFA) production rate, which yields a similar result ([93]; note that this transformation assumes that the conversion of gas production into SCFAs does not scale with BM). Gut microbes produce SCFAs, and their concentration in rumen contents of African ruminants has been shown to decrease with increasing BM in two independent datasets (Fig. 3c). The ratio of the two major SCFAs, propionate∶acetate, which decreases with a decreasing proportion of easily digestible carbohydrates and increasing proportion of fibre, decreased with increasing BM in the same two datasets, with large browsers as outliers (Fig. 3d). For the same reasons mentioned above, these comparisons are necessarily limited to foregut fermenters, i.e. mostly ruminants.

Faecal material will necessarily integrate both diet and animal effects. Estimating diet quality from faecal measures, therefore, requires a priori knowledge of factors that determine digestive efficiency: Given the finding mentioned in section 3.3 that, on a consistent diet, digestibility (which will, on that diet, only vary according to the animal factor digestive efficiency) does not scale with BM (Fig. 5e–g), any scaling of a digestibility proxy as derived from faeces in free-ranging animals will therefore necessarily indicate a scaling of diet degradability, i.e. diet quality [18]. Because herbivores rely on symbiotic gut microbes for digestion, and microbes contain high proportions of nitrogen (N), total faecal nitrogen (TFN) and metabolic faecal nitrogen (MFN, the faecal N not derived from undigested plant N) are proxies for the proportion of microbial matter in faeces; this proportion will be higher on more digestible diets [94], [95]. The principle of using TFN as a proxy for digestibility was experimentally validated in domestic cattle and sheep [19], [96], horses [97] as well as in more limited studies in wild sheep [98], deer [99], antelopes and equids [100], [101] and rodents [102]. In animals that ingest high amounts of plant secondary metabolites such as tannins, higher TFN values will reflect not only digestibility but also the fact that tannins bind protein, render it indigestible, and lead to higher faecal N excretions on lower-quality (i.e., high-tannin) diets [103]; TFN is therefore limited to animals not consuming significant amounts of tannin-containing forage. TFN has been shown to decrease with increasing BM in free-ranging African herbivores, with an outlier position of the giraffe in three datasets (Fig. 3e), corresponding to this species' high tannin intake in the wild via acacia browse [104]. At the same time, fibre contents increased in the same faecal samples (Fig. 3f). Although faecal fibre has not been validated as an indicator of diet quality, we can assume that a higher faecal fibre content represents a higher proportion of undigested plant residue and hence also a proxy for diet degradability. Recently, Steuer et al. [18] presented data on MFN that indicate that when using this proxy of diet degradability, giraffe appear as no outlier to the overall decreasing trend with increasing BM – suggesting that MFN might be more suitable than TFN to compare a wide range of herbivore species.

In summary, digestibility proxies give the strongest direct support so far for a decreasing diet quality with increasing BM in free-ranging herbivores. While many proxies in gut contents are limited in their use to ruminants, faecal indicators of diet degradability have a high potential to demonstrate variation in herbivores in general. So far, these indicators do not allow conclusions on physiological mechanisms that could bestow larger herbivores with a digestive advantage.

## Food Intake

### 4.1 Herbivores and diet quality: compensating by food intake

If we accept a decrease of diet quality with increasing BM, there are basically two options how herbivores could cope with this predicament [16].

1. If intake and metabolic requirements have the same scaling with BM, then larger animals need a higher digestive efficiency.
2. If larger animals do not achieve higher digestive efficiencies, then the scaling of intake and metabolic requirements must differ; there are three options:
  - Metabolic requirements are lower in large herbivores than in other mammals; i.e. while intake scaling is similar across mammals, metabolic scaling is lower in large herbivores.
  - Food intake is higher in large herbivores than in other mammals; i.e. while metabolic scaling is similar across mammals, intake scaling is higher in large herbivores.
  - A combination of a. and b. could apply.

Although option 1 has been traditionally used to explain large herbivore niche differentiation and diversification, little evidence exists to support it, as described in the chapters above. For option 2a, there is currently no evidence. The most comprehensive comparison of energy intake in herbivores and carnivores (though limited due to a series of assumptions) is probably that of Farlow [105], which shows overlap in the 95% CI for the scaling between the groups. The possibility that herbivores have lower levels of metabolism than vertebrate-eating carnivores has been discussed [38], but this refers to the *level* of metabolism, not its *scaling*. In the study of Capellini et al. [106] where basal metabolic rate was analysed phylogenetically, the scaling in Carnivora was not different from that of other mammalian groups. Nevertheless, the possibility that some megaherbivores have reduced metabolism, as suggested in feeding trials in hippos [107] or potentially in the particularly long gestation period of giraffes and perissodactyls [17], might deserve attention in the future.

In contrast, there is evidence for option 2b, because two independent studies (using different datasets) found that dry matter intake in large herbivores scales to a higher exponent (BM^0.84–0.90^) [16], [108] than that of mammalian metabolism (BM^0.72^) [37], [106], with confidence intervals not overlapping. Correspondingly, Bourlière [109] found that dry matter intake scaled to BM^0.72^ in 12 carnivorous and to BM^0.84^ in 12 herbivorous species. In a word, larger herbivores do not digest better, they simply eat more.

### 4.2 Does intake capacity increase with body size?

**Figure 6:**
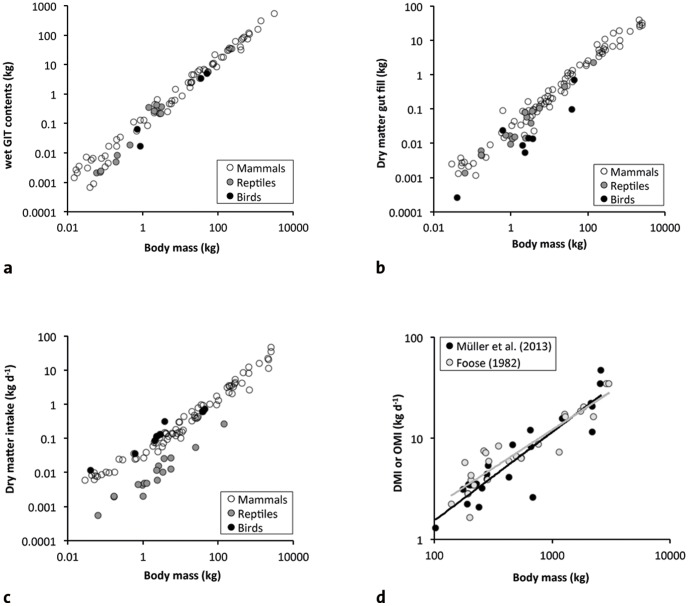
Relationships between body mass (BM) and aspects of the digestive physiology of herbivorous vertebrates. a) wet gut contents [79], [122]; note the similarity in all three vertebrae clades, with a duck species (a flying bird) as a notable outlier; b) dry matter gut contents as calculated from simultaneous passage and digestion studies [16], [81], [82]; note the similarity in the scaling of both measures of gut fill in all three vertebrate clades, with herbivorous birds falling into two categories (flying birds with lower gut fills; flightless or flight-reduced birds such as hoatzin and ostrich with gut fill as in mammals); c) dry matter intake in feeding studies in captivity [16], [81], [82]; note the generally lower intake in reptiles as compared to mammals and birds; a curvature in mammals is evident with a lower scaling in smaller and a steeper scaling in larger species; d) dry matter intake (DMI, on a variety of diets) [16] or organic matter intake (OMI, on a consistent diet) [66] in mammal herbivores >100 kg (no smaller species included in the Foose dataset); note a tendency for a lower scaling in the Foose dataset (see text) that is not significant, raising the question whether the steeper intake scaling in larger herbivores in the Müller et al. dataset is a reaction to a putative decreasing diet quality with increasing BM.

Could it be that large body size represents an advantage with respect to simply ‘eating more’? If this could be demonstrated, then the evolution of large BM might still be driven by lower diet quality. The original concept of the Jarman-Bell-principle (reviewed in [16]) stated a difference in the scaling of gut capacity as measured by wet gut contents, which scales approximately linearly (reviewed in [79]), i.e. to BM^1.0^ (Fig. 6a), and metabolic requirements (BM^0.75^). This difference was interpreted as indicating that in larger animals, more gut capacity is available per unit energy requirement. This could, in theory, also mean more leeway for larger animals in terms of food intake. Empirical tests of this concept are difficult, however, and existing data are controversial.

So far, no easily available proxy exists for intake capacity. The scaling of wet gut contents might be complicated by the possibility that moisture content of digesta increases systematically with BM [16], [83], for example to compensate for the increasing diffusion distances in the more voluminous guts of larger herbivores [71]. This would mean that the part of gut capacity that is relevant in terms of nutrient intake, i.e. dry matter gut contents (Fig. 6b), has a slightly lower scaling than one would expect based on wet gut content data. Experimental data from various herbivores in captivity indicate that no statistical difference in the scaling of intake (Fig. 6c) and dry matter gut capacity can be demonstrated [16], but nevertheless they both scale higher than metabolism in large herbivores. Yet, the fact that larger animals increase intake or gut contents more than metabolism in empirical datasets, where the diet is not controlled, such as in wet gut contents from animals taken from the wild (Fig. 6a), or in data compilations from a variety of feeding studies in captivity (Fig. 6bc) where diet quality might for example systematically differ with BM as in the wild (as suggested by faecal N data for zoo animals in [94]), might simply represent an actual condition where larger animals need to compensate for lower diet quality more distinctively, *and not that smaller animals cannot do so*. They simply might not have to do so under the conditions where the data were generated – not in the wild, because they can select higher-quality diets, nor in captivity, where they might be fed such diets. Comparing the scaling of intake from a compiled dataset and from a dataset where a consistent diet was fed to large herbivores (Fig. 6d) could suggest this possibility: on the consistent diet, the scaling of intake is numerically lower (i.e., smaller animals eat more) than in the compiled dataset (note that the data scatter is too high and the sample size too low for statistical significance).

Additionally, selected examples could indicate that differences in intake capacity can occur between species of the same body size range, which would make this attribute rather independent from BM but a characteristic of a specific *bauplan*. Apparently, hippos are much more constrained in their capacity for high food intake, in contrast to elephants [110]. On the other end of the BM range, rabbits (*Oryctolagus cuniculus*) are known to have difficulties to maintain condition on low-quality roughage (e.g. [111]), whereas this is not evident in guinea pigs (*Cavia porcellus*), which in comparison feed less selectively and have higher gut fills [112]. Selectively including one or the other species in a comparative dataset could thus yield different conclusions as to effects of BM on intake capacity. To date, current data cannot be reliably used to prove or exclude the possibility that larger body size is linked to a disproportionately higher intake capacity.

### 4.3 Instantaneous or anticipatory compensation of low diet quality and fasting endurance

Appealing as the concept that larger animals compensate for lower diet quality by a generally increased intake may be, intraspecific data do not unanimously indicate such a strategy. In contrast, larger herbivores typically show a strategy that could be called ‘anticipatory’, with a higher food intake on higher quality diets, and a reduction in food intake on lower quality diets [113]. In particular, reasons for a reduction of intake on lower quality diets remain to be investigated. Traditionally, the reason for this has been sought in a dichotomy between ruminants, which are supposed to be physically limited in their intake capacity by low-quality forage because of rumen physiology, and hindgut fermenters, which should not be thus constrained (reviewed in [113]). Empirical data, however, do not support this dichotomy, and hindgut fermenters also appear to reduce food intake on low quality forages. Reasons for the reduction of food intake on lower quality diets therefore might rather be related either to gut fill limitations on lower quality forages that apply to all herbivores, or to higher endogenous and metabolic losses on such diets. Only in some smaller herbivores (who also practice coprophagy, which reduces endogenous/metabolic losses) was an ‘instantaneous’ compensation - increasing food intake with lower diet quality – observed [113]. This difference matches the higher capacity for resource accretion as body (adipose) tissue and the corresponding higher fasting endurance in larger animals [114]–[116]. In addition to a strategy of accreting body reserves, larger animals are also more likely to adopt a strategy of migration to ensure high forage quality [117]. In contrast, smaller animals are mostly unable to evade their habitat in times of lower food quality, and need to resort either to energy saving via a reduction in metabolism, such as hibernation, or to food caching, or have to live on the lower quality food. Fasting endurance is an important benefit bestowed by large body size [31], but is notably not a direct effect of alterations in digestive physiology.

## Relevance for Dinosaur Gigantism

What conclusions do these physiological reflections allow for giant dinosaur herbivores? From comparisons with extant representatives of putative dinosaur food plants [118], there do not appear to be major differences in the fermentation characteristics between dinosaur forage and important extant mammal herbivore forage like browse [51], [89]. Possible differences in nitrogen content [14], [89] and plant secondary compounds cannot be considered as drivers of directed body size evolution, as explained in sections 3.1 and 3.2.

**Figure 7:**
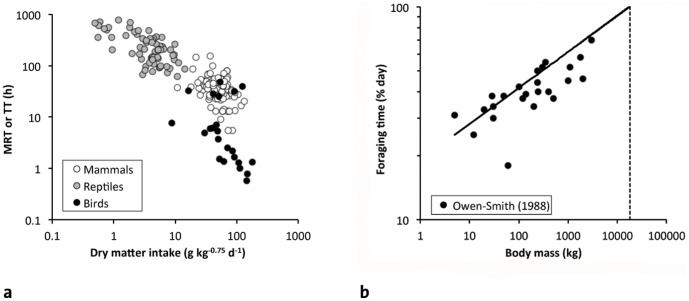
Relationships between aspects of the digestive physiology of herbivorous vertebrates. a) the relative food intake (per unit metabolic body weight) and the passage of digesta through the gastrointestinal trat (measured as mean retention time MRT or, in the case of some reptiles, as transit time TT) [81], [112]; note that species/individuals with a higher food intake have shorter retention times; note that flying birds show a similar relationship on a lower level, potentially due to their smaller gut capacity (cf. Fig. 6b); b) body mass and foraging time for hindgut fermenters and ruminants [42] (regression given for hindgut fermenters; extrapolation to 100% of the day yields an upper BM limit of app. 18 tons).

Sauropod dinosaurs are peculiar due to the absence of a particle size reduction mechanism (chewing teeth or gastric mill) [119]. Given indications for a high level of metabolism in sauropods due to their fast growth [1], we would thus expect a food intake level comparable to mammals (Fig. 6c) combined with digesta particle sizes comparable to reptiles (Fig. 5a). The faster digesta passage, i.e. the shorter retention times in mammals as compared to reptiles are usually interpreted as possible due to the higher degree of particle size reduction, because smaller particles can be fermented faster by microorganisms [82], [120], and a compensation between retention time and chewing efficiency is also evident in mammals [87], [121]. Therefore, we would expect retention times in sauropod dinosaurs to be more similar to those of reptiles (Fig. 5c), to efficiently digest the non-comminuted digesta. Because of a link between food intake and retention time (times are shorter at higher intake levels) (Fig. 7a) [81], [112], a plausible mechanism to maintain a reptile retention time at a mammalian food intake would be to have higher gut capacities than reported for both reptiles and mammals (Fig. 6ab). Actually, a comparison of the reconstructed volume of the coelomic cavity of a sauropod with the volume of the organs within that cavity suggest sufficient spare capacity of that coelomic cavity to accommodate disproportionately large guts [122]. Based on this logic, we would expect non-chewing herbivorous dinosaurs with a high metabolism, such as sauropods, to have comparatively larger coelomic cavities than chewing herbivorous dinosaurs, such as ornithopods. This hypothesis awaits testing. Another hypothesis, namely ontogenetically reduced metabolic rates in adult sauropods [123], provides a convenient *ad hoc* explanation yet is more difficult to test.

Allometries related to chewing and particle size reduction can potentially indicate that the absence of chewing in sauropods is a condition that does not necessarily drive but facilitate gigantism [1], [123]. An important part of mammalian foraging time is dedicated to the act of (ingestive) mastication [21]. According to the scaling of foraging time [42] (Fig. 7b), mammal nonruminant herbivores above a BM threshold of 18 tons would require more than 24 h of foraging time per day. Evidently, the database for this allometry consists of few species, and the magnitude of scaling would change distinctively if only a few values were added or existing ones modified. Nevertheless, it is intriguing that none of the largest chewing herbivores, neither the largest mammal, the *Indricotherium* [124], nor the large ornithischians with their impressive chewing dentition [125] – such as *Shantungosaurus* [126], surpass this mass threshold [127]. The interpretation appears attractive that herbivores, once they evolved the very efficient adaptation of mastication, were generally prevented from evolving giant body size because this would have necessitated a secondary loss of mastication. Thus, it seems that a primitive feature of sauropods – the absence of mastication – allowed them to enter the niche of giants. It remains to be seen whether findings of ornithischians beyond the BM threshold do or do not show characteristics of a chewing dentition.

Finally, with respect to another digestive side-effect, as long as the few existing indications that herbivorous birds, which are closer related to dinosaurs, have a dramatically lower methane production than mammals (reviewed in [81]) are not refuted, extrapolations on the production of methane by dinosaur faunas based on mammal data (e.g. [128]) should be viewed with scepticism.

To conclude, we think that existing data suggest that other putative advantages of large body size [1] are more promising candidates for the explanation of the evolution of gigantism than digestive physiology.

## Outlook on Outliers: Which Rule Do Exceptions Prove?

In mammals, birds and reptiles, small-bodied herbivore species have been described that appear to ‘break’ or ‘bend’ the ‘ecophysiological rule’ that small BM must be linked to high-quality diets [83], [129], [130]. What do these outliers tell us? The traditional approach to such species is to identify physiological mechanisms that allow them to use these unexpected resources. We want to propose a different scenario, based on the logic outlined in section 1.2 that a ‘higher mass-specific metabolic requirement’ in itself has no explanatory power. Rather than using a physiological argument, we suggest an ecological one.

If we accept the theoretical possibility that animals of any size can use diets of any quality, given that these diets are available in sufficient quantity and accessible packages, we will, in terrestrial systems, still end up with a dichotomy of choices: because of forage abundance and the impracticability of selective feeding, larger herbivores are (mostly) confined to low quality diets. Small herbivores, however, theoretically have both options – because of their smaller absolute requirements, and their smaller feeding apparatus, they can use both, high and low quality diets. Smaller animals might be excluded from a certain range of plants or plant parts because of physical limitations, especially in the cropping of larger-diameter lignified tissues (stems and twigs); yet, adaptations to such diets exist, as in the gnawing feeding style of rodents [131]. Note that this is a physical argument related to the mechanics of feeding, not to digestive physiology.

Rather than suggesting that small herbivores *cannot* use the lower diet quality, we could ask – why *should* they? Given their opportunity to use the higher-quality resource, it appears plausible that they would focus on the latter, and potentially even lose, over evolutionary time, adaptations to cope with the former – *not because of a body size-driven physiological necessity, but because of ecological opportunity*. Exploring this scenario, and testing it against patterns actually observed, could represent a promising approach to understand ecological and evolutionary patterns in herbivores. It might also allow to integrate the under-emphasized outlier position of extant megaherbivores in many datasets presented in this review, and link herbivore nutritional ecology by unifying concepts of biomass availability and food accessibility to that of omnivores and carnivores. Shifting the focus from a putative link with digestive physiology that might, in many cases, rest on a rhetoric misunderstanding, to an ecological approach, might finally yield better theories about the relationship of diet and body size that match actually observed patterns both in extant herbivores and in the fossil record (e.g. [132]).
